# Acute and Cumulative Effects of Haze Fine Particles on Mortality and the Seasonal Characteristics in Beijing, China, 2005–2013: A Time-Stratified Case-Crossover Study

**DOI:** 10.3390/ijerph16132383

**Published:** 2019-07-04

**Authors:** Yi Li, Canjun Zheng, Zhiqiang Ma, Weijun Quan

**Affiliations:** 1 State Key Laboratory of Severe Weather & Key Laboratory of Atmospheric Chemistry of CMA, Chinese Academy of Meteorological Sciences, Beijing 100081, China; 2 Chinese Center for Disease Control and Prevention, Beijing 102206, China; 3 Institute of Urban Meteorology, China Meteorological Administration, Beijing 100080, China

**Keywords:** time-stratified, case-crossover, haze, PM_2.5_, mortality, seasonal

## Abstract

We observed significant effects of particulate matter (PM_2.5_) on cause-specific mortality by applying a time-stratified case-crossover and lag-structure designs in Beijing over a nine-year study period (2005–2013). The year-round odds ratio (OR) was 1.005 on the current day with a 10 μg/m^3^ increase in PM_2.5_ for all-cause mortality. For cardiovascular mortality and stroke, the ORs were 1.007 and 1.008 on the current day, respectively. Meanwhile, during a lag of six days, the cumulative effects of haze on relative risk of mortality, respiratory mortality and all-cause mortality was in the range of 2~11%. Moreover, we found a significant seasonal pattern in the associations for respiratory mortality: significant associations were observed in spring and fall, while for all-cause mortality, cardiovascular mortality, cardiac and stroke, significant associations were observed in winter. Moreover, increasing temperature would decrease risks of mortalities in winter taking fall as the reference season. We concluded that in summer, temperature acted as a direct enhancer of air pollutants; while in winter and spring, it was an index of the diameter distribution and composition of fine particles.

## 1. Introduction

Haze is one kind of serious air pollution caused by the accumulation of fine particulate matter (PM_2.5_) in the atmosphere. It could be attributed to air pollutants emission to the lower atmosphere from fossil fuel combustion or construction and other causes concurrent with unfavorable meteorological diffusion conditions. Air pollutants include polluted gases (sulfur oxides, oxynitride, carbon oxide, and hydrocarbon compounds) and particulate matters (PM_2.5_) from both industrial and natural sources such as sand-dust, bacteria and spore [1]. In nearly 20 years, severe haze events have been showing an increasing trend in China. For example, in 2013, several extreme haze events occurred over northern and eastern China [2,3]. Also in the year 2013, an extremely severe haze incident occurred which lasted for seven days in January. In this incident, the hourly PM_2.5_ concentrations in many cities exceeded the upper limits of the Air Quality Index. Frequent hazy weather, which has lots of hazardous material, may have adverse effects on human health as well as the climate [3,4,5,6,7,8].

Beijing is the largest megacity in northern China as well as one of the areas with the most severe haze incidents. The number of annual average haze events in Beijing is about 1.5 times higher than the national average (31.7 days to 20 days, 1961–2013) [1]. Meanwhile, the frequency of haze events shows an increasing trend (9.7 days/10years) [1]. However, studies on the health impacts of haze in Beijing are very limited. As the main toxic component in haze, the association between fine inhalable particles and cause-specific mortalities has been investigated as well as the role of temperature in Beijing [9,10,11,12,13,14], and these studies showed that chronic exposure to fine particulate air pollutants was associated with increased relative risk of respiratory mortality. Most of the literature focuses on the acute effects of air pollutants on mortality; however, fine particles may increase the risk for adverse health when exposure occurs over a longer duration. As haze often lasts for several days, the effects of PM_2.5_ on morbidity/mortality might delay or last for longer periods, and cumulative effects could be expected [15]. 

Several studies [16,17] have explored longer-term effects and gave out positive conclusions. More importantly, the cumulative effects of the lag combined with temperature and the seasonal characteristics caused by varying emissions and atmospheric reactions have not been explored. Considering the lag and accumulative effects of haze and the difference between haze particles and non-haze particles in composition and size distribution, the role of temperature in PM_2.5_ effects on health outcomes might be different. 

In this study, we used a case-crossover and non-linear lag model with suitable structures to assess the acute and cumulative effects of PM_2.5_ particles on mortality. More importantly, we aimed to explore how temperature/season modified the effects of PM_2.5_ with varying emissions and size distribution. Our study goal was to assess the impacts of hazy fine particles on human mortality in northern China and to discuss the seasonal modification on PM_2.5_ effects.

## 2. Materials and Methods

### 2.1. Study Area

**Figure 1:**
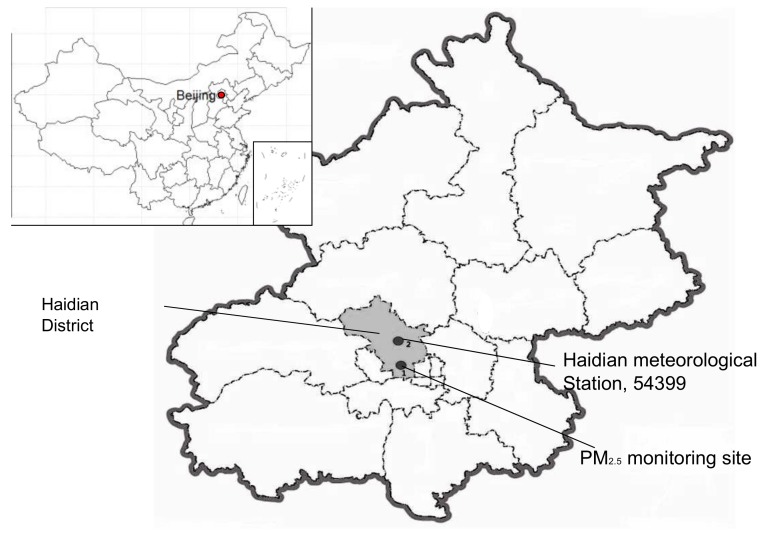
Study area. Shaded part indicates Haidian District.

We conducted the study in an urban area of Beijing (Haidian District, see Figure 1), China, from 1 January 2005, to 31 December 2013 (3287 days). Haidian District is situated in northwest Beijing, covers an area of 430 square kilometers, and has a population of more than 3 million. It stretches from latitude 39°53′ N to 40°09′ N and from longitude 116°03′ E to 116°23′ E. Haidian spans approximately 30 kilometers from north to south and 29 kilometers from east to west.

Haidian District lies in a continental monsoon region in a warm temperature zone and has an average yearly temperature of 12.3 °C. It is windy in spring and dry and cold in winter [13].

### 2.2. Data Collection

The data sets consisted of concurrent daily time series of health outcomes, meteorological factors and air pollution collected in Haidian District. We obtained mortality data from the Chinese Center for Disease Control and Prevention.

Daily meteorological variables (such as temperature, visibility, relative humidity, pressure, wind speed, and wind direction) were recorded from the China Meteorological Administration (CMA). Daily PM_2.5_ was recorded every 5 minutes by tapered-element oscillating microbalances (TEOM, model1400a, Rupprecht and Patashnick; Thermo Electron, East Greenbush, NY). Atmospheric visibility, gaseous pollutants (SO_2_, NOx, carbon monoxide (CO), ozone (O_3_)) and meteorological variables (such as temperature, planetary boundary layer heights, relative humidity (Rh), pressure, wind speed, and wind direction) were observed simultaneously [18]. The collocated gaseous species, including CO, SO_2_, NOx and O_3_, were observed by a various gas analyzer (Thermo Scientific Co., USA).

According to the definition by the Chinese Meteorological Administration (CMA), a haze event is defined by the following conditions: visibility lower than 10 km and relative humidity (Rh) lower than 90%. In Beijing, fine particulate matter is the primary cause of haze events. In this study, after considering the CMA definition and the measured data (air pollutants and visibility), we defined a day with a PM_2.5_ daily mean concentration lower than 50 μg/m^3^ as a non-haze day (visibility >10 km), a PM_2.5_ daily mean concentration in the range of 50~100μg/m^3^ as a light haze day (8 km < visibility < 10 km), a PM_2.5_ daily mean concentration in the range of 100~200μg/m^3^ as a medium haze day (5 km < visibility < 8 km)and a PM_2.5_ daily mean concentration >200 μg/m^3^ (visibility <5 km) as a heavy haze day. 

To test seasonal variations of haze effects on mortality, all data were divided into four seasons: spring (from March to May), summer (from June to August), fall (from September to November) and winter (from December to February).

Mortality data of Haidian District was from Chinese Center for Disease Control and Prevention (China CDC) including deaths due to diseases (ICD-10 code: I00-I99), respiratory diseases (J00-J99), stroke (ICD-10 code: I60-I69), digestive diseases (ICD-10 code: k00-I93), genitourinary diseases (ICD-10 code: N00-N99), cardiac diseases (ICD-10 code: I00-I09, I20-I52) and all-cause mortality (A00-R99). From 1 January 2005, to 31 December 2013, 3287 days were recorded.

### 2.3. Statistical Methods

A time-stratified case-crossover design was used to investigate the acute associations between PM_2.5_ and cause-specific mortality, and we used conditional logistic regression to calculate odds ratio (OR) for PM_2.5_ correspond to a 10 µm/m^3^ increase. In case–crossover study design, the idea is to compare “case” days when deaths occurred with control days to look for differences in exposure that might explain differences in the number of cases. There are two main parts to a case–crossover analysis: (1) matching the controls days to the case days, (2) performing the conditional logistic regression to calculate the odds ratio for cases compared with controls for a unit increase in exposure. In our study, we chose the unit increase as 10 µg/m^3^. We used 35-day stratum length with an exclusion period of three days and controlled for day of the week. The case-crossover study design inherently controls for factors that do not vary within person (e.g., age, sex, genetics) and adjusts for confounding by longer term trends and meteorological factors. Current day was designated for the day that death was reported. The stratum length was 35 days and the exclusion period was three days. DOW (day of the week) was also considered as a dummy variable.

In our study, we controlled more rigidly for temperature to assess the effects of PM_2.5_ more accurately. To achieve this goal, we set temperature as an independent confounder and selected control days within a same temperature range (in our study this range was 2 °C) as the case day. So shape of the non-linear association between temperature and mortality risk is not important. R software [19] and the “season” package [20,21] were used to perform the analysis.

In this study, we also applied a distributed lag non-linear model (DLNM) to describe the cumulative health effects of PM_2.5_ with the “dlnm” package in R [22]. This model class is based on the definition of a cross-basis, a bi-dimensional space of functions describing the association along the spaces of predictor and lags. The main advantage of this method is that it allows the model to contain a detailed representation of the time-course of the exposure–response relationship, which in turn provides an estimate of the overall effect in the presence of delayed contributions. In this methodology, estimated relationships allow for a temporal structure of dependency. This is useful because in environmental epidemiology studies, a specific exposure event often affects health outcomes for a period of time beyond the actual event and cumulative effects could be expected. We used a linear DLNM constrained to a 5-day lag period to effectively capture the overall effects of haze. 

All statistical tests were two-sided, and *p* < 0.05 was considered statistically significant.

### 2.4. Sensitivity Analysis

We assessed the robustness of the DLNM model and logistic regression model via sensitivity analysis, replacing the mean daily temperature with maximum and minimum daily temperature. We also changed stratum lengths. To analyze the possible contribution of SO_2_ and NO_2_, we input these two pollutants into these two models.

## 3. Results

### 3.1. Descriptive Statistics of Data

Table 1 provides summary statistics of the variables in our study. Daily mean temperature ranged from −14.1 °C to 32.8 °C, relative humidity ranged from for 7% to 100% and PM_2.5_ ranged from 20.02 µg/m^3^ to 301.70 µg/m^3^. There was a total of 8247 respiratory deaths, 34,533 cardiovascular deaths, 13,083 strokes, 2211 metabolic deaths, 19,996 cardiac deaths, 733 genitourinary deaths and 70,614 total all-cause deaths during the study period.

### 3.2. Estimated Effects of PM_2.5_

Table 2 shows that for year-round effect, PM_2.5_ had some significant associations on the current day: with a 10 µg/m^3^ increase, ORs were 1.005 (95% confidence interval (CI): 1.001–1.008), 1.006 (95% CI: 1.002–1.010) and 1.008 (95% CI: 1.001–1.014) for all-cause mortality, cardiovascular mortality and stroke, respectively. For stroke, PM_2.5_ also had significant effects on lag 1 day: with a 10 µg/m^3^ increase, OR was 1.005 (95% CI: 0.999–1.010). No significant effect was found between PM_2.5_ and respiratory mortality or cardiac. 

As for spring and fall, significant effects of PM_2.5_ only showed on respiratory mortality. For spring on lag 1 day, with a 10 µg/m^3^ increase of PM_2.5_ concentration, OR was 1.039 (95% CI: 1.000–1.078). For fall, with a 10 µg/m^3^ increase of PM_2.5_ concentration, ORs was 1.044 (95% CI: 1.015–1.075) and 1.028 (95% CI: 1.000–1.056) for current day and lag 3 day, respectively.

For summer, PM_2.5_ had significant effects on cardiac for lag 1 day on stroke for current day: with a 10 µg/m^3^ increase of PM_2.5_ concentration, ORs were 1.022 (95% CI: 0.997–1.048) and 1.033 (95% CI: 1.001–1.066), respectively. 

As for winter, PM_2.5_ had significant effects on all-cause mortality, cardiovascular mortality and cardiac. For all-cause mortality, ORs were 1.026 (95% CI: 1.011, 1.042), 1.017 (95% CI: 1.002, 1.032), 1.021 (95% CI: 1.005, 1.036) and 1.030 (95% CI: 1.015, 1.044) on current day, lag 1 day, lag 3 day and lag 4 day, respectively. For cardiovascular mortality, ORs were 1.031 (95% CI: 1.013, 1.050), 1.025 (95% CI: 1.007, 1.044) and 1.037 (95% CI: 1.019, 1.055) on current day, lag 1day and lag 4 day, respectively. For cardiac, ORs were 1.028 (95% CI: 1.003, 1.052) and 1.040 (95% CI: 1.016, 1.065) on current day and lag 4 day, respectively. 

### 3.3. Cumulative Effects

According to studies [19], duration time of haze events in Beijing ranged from 1–6 days. In “long haze” events, fine particles would form with an accumulation mode and the concentration would gradually increase to a high value in several days. 

**Figure 2:**
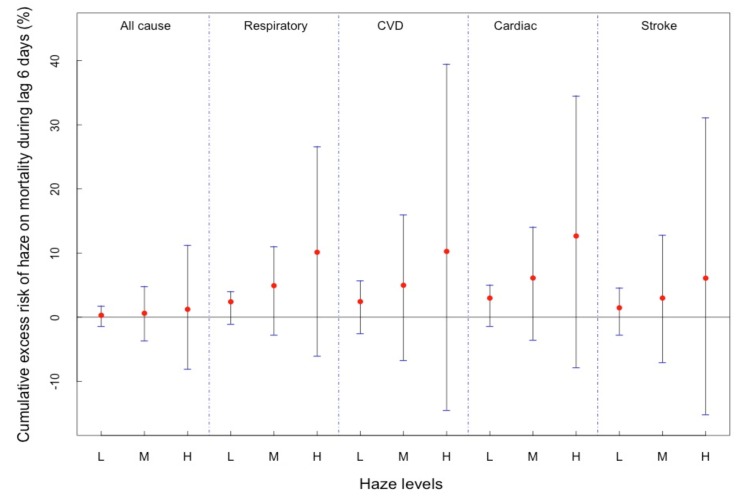
Cumulative excess risk of haze particles on mortality over lag 0~5 days. Notes: L: Light haze (an increase of PM_2.5_ concentration of 50 μg/m^3^). M: Medium haze (an increase of PM_2.5_ concentration of 100 μg/m^3^). H: Heavy haze (an increase of PM_2.5_ concentration of 200 μg/m^3^). Bars represent the 2.5% and 97.5% CI.

Figure 2 shows the cumulative effects. We discovered that over 5 days, the cumulative effects of haze on cardiovascular mortality risks were 2.4% (95% CI: −1.02%~4.01%), 4.91% (95% CI: −2.70%~11.07%) and 10.12% (95% CI: −5.99%~26.64%) for light haze, medium haze and heavy haze, respectively. For respiratory mortality, the cumulative risks were 2.43% (95% CI: −2.57%~5.70%), 4.98% (95% CI: −6.70%~15.94%) and 10.25% (95% CI: −14.46%~39.49%), respectively. For cardiac mortality, the cumulative effects were 2.97% (95% CI: −1.35%~5.07%), 6.11% (95% CI: −3.55%~14.10%) and 12.66% (95% CI: −7.80%~34.57%), respectively. For all-cause mortality, the values were 0.30% (95% CI: −1.39%~1.79%), 0.61% (95% CI: −3.67%~4.84%) and 1.23% (95% CI: −8.08%~11.23%).

### 3.4. Seasonal Characteristics of PM_2.5_

**Figure 3:**
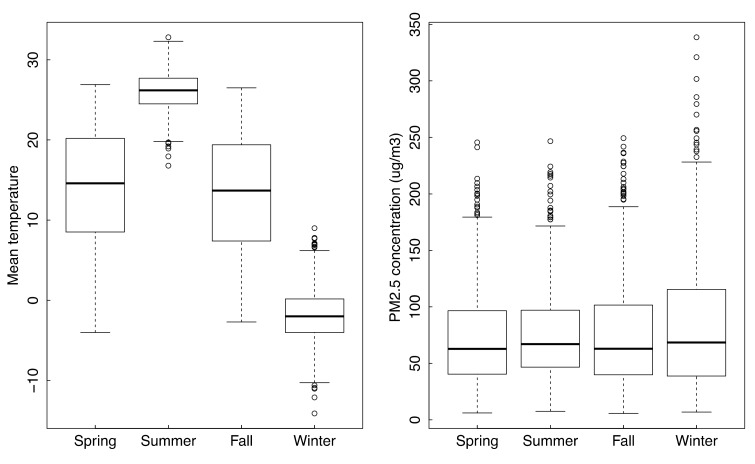
Boxplot of seasonal mean temperature and PM_2.5_ concentration.

**Figure 4:**
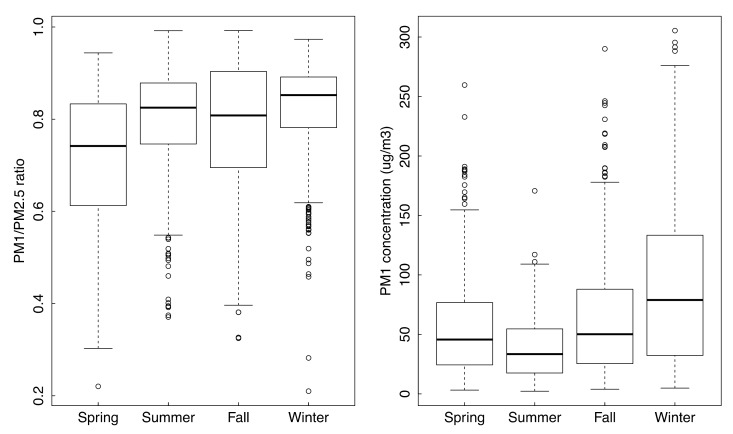
Boxplot of PM_1_/PM_2.5_ ratio and PM_1_ concentrations in four seasons.

Although PM_2.5_ concentrations were significantly different among the four seasons, they did not fluctuate too much (Figure 3). However, there were significant seasonal variations (*p* < 0.01) of PM_1_/PM_2.5_ ratios and concentrations of PM_1_ (Figure 4).

### 3.5. Direct Effects of Temperature

Table 3 shows odds ratios for daily cause-specific mortality for 1 degree rise in ambient temperature in the three seasons taking fall as the reference season. The odds ratios in winter were all smaller than 1, meaning that in winter, increasing temperature would decrease the risk of all cause-specific mortality. No significant effect of temperature was seen in spring and summer. 

### 3.6. Sensitivity Analysis

To analyze the possible contribution of SO_2_ and NO_2_, we input these two pollutants into the model. Estimates of PM_2.5_ changed very little after controlling for SO_2_ and NO_2_. We found that the effect of haze did not significantly change after controlling for SO_2_ and NO_2_.

Sensitivity analyses indicated that degree of freedom and length of stratum did not significantly change the findings. Also, we found very little change after we replaced mean daily temperature with maximum and minimum daily temperature.

## 4. Discussion

In this study, we found significant impacts of PM_2.5_ on cause-specific mortalities in winter for cardiovascular mortality and all-cause mortality on current day and lag 1 day. Also, we found significant associations between respiratory mortality and PM_2.5_ in spring and fall, on current day and lag 1 day. In above associations, increasing PM_2.5_ concentration would increase odd ratios of cause-specific mortality. Moreover, the cumulative risks of cardiovascular mortality, respiratory mortality and all-cause mortality increased with an increase of PM_2.5_ concentration.

We also found significant seasonal patterns in these associations. As temperature is thought to be the most important characteristic of seasonal weather and the most important meteorological confounder or modifier in the association between air pollution and health effects, we explored the impacts of temperature as well. We found that significant effects of temperature appeared only in winter if we took fall as the reference season: increasing temperature would decrease the odds ratio of mortality. Moreover, except for respiratory mortality, all the estimates of associations in winter were larger than those in other seasons. These results suggested that low temperature enhanced the effects of PM_2.5_ on mortality. Being different from our work, enhancement of acute mortality effects of fine particles by temperature in summer was discovered in some Chinese cities such as Wuhan [23] and Tianjin [24]. The reason might be that extremely high temperature could make a person more allergic to toxic agents like PM_2.5_. Moreover, on warm and hot days, people might be more likely to go outside or open windows, and thus increase exposure to fine particles.

On the other hand, seasonal pattern in PM_2.5_-mortality associations could be different according to geographic characteristics. For example, according to one research work [25] in the US, for cities in cold regions, both heat and cold might increase mortality risk. In cities in warm regions, only heat was observed to lead to significant effects on mortality. In Guangzhou (23°20″, 113°30″), a subtropical city in south China, PM_2.5_ was found to have significant effects in cold season. The “inconsistency” might come from people’s different exposure mode during warm and cold seasons.

In our study, we found that the most severe effects of PM_2.5_ on health outcomes occurred in winter. Almost all of the ORs in winter were significantly higher than those for the entire year or other seasons for all-cause mortality, cardiovascular mortality and stroke. Besides that, increasing temperature would decrease the odd ratio of mortality in winter; the reason that winter had a higher and more significant association might due to the different diameter distribution and composition of PM_2.5_ from the other three seasons. We think season/temperature was not the main directly affecting factor but represented an index of PM_2.5_ emission source, diameter distribution and composition. The following was one plausible explanation for this opinion.

It should be noted that the different effects of haze and non-haze weather are not only attributable to the concentration of fine particulate matters. Instead, their composition and diameter distribution are the primary factors that affect health outcomes [26]. In Beijing, the ratios of PM_1.0_/PM_10_ during haze days were greater than 0.47, while during non-haze days the ratios were less than 0.42 [27]. Thus, people are potentially exposed to more fine particles and toxic compositions on haze days. Previous investigations on fine inhalable particles in Beijing have confirmed that fine particles that could induce oxidative stress were mainly concentrated in 0.56–1.0μm diameter particles [27], implying that fine particles have more oxidative capacity.

The dominant sources and formation mechanisms of PM_2.5_ vary greatly among the four seasons, as do the chemical composition and fractions in different size distributions. In Beijing, coal burning is one of the most important sources of ambient particulate matter, especially in wintertime, and this plays a major role in the formation of haze. Concentrations of toxic chemical components in fine particles were higher in winter. Moreover, according to some research [28], the ratios of PM_1.0_/PM_10_ were highest in winter compared to the other three seasons (0.63 in winter and 0.53 in other seasons), which means more severe fine particle pollution occurs in winter. According to our measurements, the mean ratios of PM_1.0_/PM_2.5_ of the four seasons were 0.71, 0.79, 0.78 and 0.82 of spring, summer, fall and winter, respectively. There were significant differences among the four seasons (*p* < 0.01, please see the Supplementary Materials, Table S1) and the highest value appeared in winter. As some studies have reported, smaller particles are easier to deposit in the deep respiratory tract and thus are more likely to enter the bloodstream and affect various bodily systems [29,30].

The PM_2.5_ concentrations and size distributions did not fluctuate too much among the four seasons on non-haze days, while chemical components of PM_2.5_ were sharply different on haze days among the seasons. This indicated that seasonal variations in were most significant on haze days. In Beijing, haze mostly occurs in winter and this might be the main reason that PM_2.5_ had the most significant effects in this season.

Compared with those on cause-specific and all-cause mortality, PM_2.5_ had the most significant impacts on cardiovascular mortality. This is congruent with most studies about the fine particles (PM_2.5_/PM_10_) effects on health [31,32,33]. Moreover, health outcomes had different seasonal characteristics. In this study, we found that in spring and fall, PM_2.5_ had the most significant association with respiratory mortality, unlike that of cardiovascular mortality. The reason might be that spring and fall has the highest concentrations of respiratory-relative allergens such as pollen or other pathogenic microorganisms compared to the other seasons. Moreover, in Beijing, these allergens often begin to be active in spring [7]. Bioaerosol is one kind of allergen that could induce or aggravate allergic reactions in the respiratory system, especially for people who already have respiratory diseases [34]. Studies also showed that concentrations of some respiratory allergens would increase with an increase of fine particles in haze events [35] due to the transportation function of fine particles. Thus, season should be an important factor and have important correlations with the concentration of bioaerosols. The diameters of respiratory-relative bacteria and fungi in spring and fall are significantly smaller than those in the other two seasons, and they are easier to deposit deeply into the respiratory system [36]. 

## 5. Conclusions

In this study, we estimated the cumulative risk as well as lag-specific estimates of haze fine particles on cause-specific mortalities with case-crossover design and lag models. We also explored the seasonal characteristic of PM_2.5_ effects on health outcomes. We concluded that haze fine particles had significant impacts on cause-specific mortalities in Beijing, and these impacts exhibit strong seasonal characteristics.

## Figures and Tables

**Table 1:** Summary of the current day’s particulate matter (PM_2.5_) concentrations, 24-h mean temperature and mortality time series data.

Variables	Mean	Minimum	25th	50th	75th	Max
PM_2.5_ (µg/m^3^)	78.01	20.02	42.6	65.94	103	301.7
Temperature (°C)	12.93	−14.1	2	14.3	23.5	32.8
Relative humidity	55.5	7	39	57	71.75	100
Respiratory diseases	2.47	0	1	2	3	11
Endocrine and metabolic	1.49	1	1	4.27	2	9
Cardiovascular diseases	9.58	1	7	9	12	24
Genitourinary	1.14	1	1	1	1	4
Total death	21.64	4	17	21	26	49

**Table 2:** Odds ratios (ORs, 95% confidence interval (CI)) for daily cause-specific mortality for a 10 μg/m^3^ increase in of PM_2.5._

Season		All-Cause	Cardiovascular	Respiratory	Cardiac	Stroke
Whole year Spring	lag 0	1.005 (1.001, 1.008) **	1.006 (1.002, 1.010) **	0.996 (0.988, 1.004)	1.002 (0.997, 1.007)	1.008 (1.001, 1.014) *
	lag 1	0.997 (0.994, 1.000)	0.998 (0.995, 1.002)	0.999 (0.992, 1.006)	0.995 (0.990, 1.000)	1.005 (0.999, 1.010) *
	lag 1	0.999 (0.981, 1.017)	1.001 (0.979, 1.023)	1.039 (1.000, 1.078) *	1.000 (0.970, 1.030)	1.000 (0.965, 1.034)
Summer	lag 0	0.993 (0.980, 1.005)	0.992 (0.977, 1.007)	1.012 (0.974, 1.051)	1.009 (0.984, 1.035)	1.033 (1.001, 1.066) *
	lag 1	0.989 (0.976, 1.001)	0.996 (0.981, 1.011)	1.005 (0.969, 1.042)	1.022 (0.997, 1.048) *	1.016 (0.985, 1.047)
Fall	lag 0	0.997 (0.986, 1.008)	0.998 (0.985, 1.011)	1.044 (1.015, 1.075) **	1.001 (0.986, 1.017)	1.004 (0.983, 1.025)
	lag 3	0.997 (0.986, 1.007)	0.989 (0.977, 1.002)	1.028 (1.001, 1.056) *	0.996 (0.980, 1.013)	1.014 (0.991, 1.036)
Winter	lag 0	1.026 (1.011, 1.042) **	1.031 (1.013, 1.050) **	0.996 (0.966, 1.027)	1.028 (1.003, 1.052) *	1.004 (0.975, 1.034)
	lag 1	1.017 (1.002, 1.032) *	1.025 (1.007, 1.044) **	0.975 (0.946, 1.005)	1.015 (0.992, 1.039)	1.017 (0.989, 1.046)
	lag 3	1.021 (1.005, 1.036) **	1.014 (0.995, 1.032)	1.004 (0.974, 1.036)	1.011 (0.987, 1.035)	1.001 (0.972, 1.030)
	lag 4	1.030 (1.015, 1.044) **	1.037 (1.019, 1.055) **	1.006 (0.976, 1.038)	1.040 (1.016, 1.065) **	1.016 (0.986, 1.046)

* *p* < 0.05; ** *p* < 0.01.

**Table 3:** Odds ratios for daily cause-specific mortality for 1 degree rise in ambient temperature in the three seasons #.

Health Outcomes	Season	OR	Confidence Interval		*p*-Value
		2.50%	97.50%
All-cause mortality	spring	1.00	1.00	1.05	0.88
	summer	1.00	0.99	1.01	0.13
	winter	0.98 *	0.97	0.89	<0.01
Cardiovascular mortality	spring	1.00	0.99	1.05	0.66
	summer	1.00	0.99	1.00	0.04
	winter	0.98 *	0.97	0.94	<0.01
Respiratory mortality	spring	1.00	0.99	1.17	0.860
	summer	1.00	0.99	1.08	0.670
	winter	0.97 *	0.94	0.97	0.030
Cardiac	spring	1.00	0.99	1.11	0.65
	summer	1.00	0.99	1.04	0.54
	winter	0.98 *	0.96	0.92	<0.01
Stroke	spring	1.00	0.99	1.08	0.64
	summer	1.00	0.99	1.05	0.36
	winter	0.98 *	0.96	0.98	0.03

# Fall was the reference season; * Significant.

## Supplementary Materials

The following are available online at https://www.mdpi.com/1660-4601/16/13/2383/s1: Table S1 Differences of PM_1_, PM_2.5_ concentration (ug/m3) and PM_1_/PM_2.5_ ratio of the four seasons.
