# Supplementary material for: Acute and Cumulative Effects of Haze Fine Particles on Mortality and the Seasonal Characteristics in Beijing, China, 2005–2013: A Time-Stratified Case-Crossover Study
Source: Int J Environ Res Public Health. 2019 Jul 4;16(13):2383. doi: 10.3390/ijerph16132383 (PMC6650878; doi:10.3390/ijerph16132383)
Supplement: Supplementary file 1 [file ijerph-16-02383-s001.pdf]

Table S1. Differences of PM_1, $PM_{2.5}$ concentration (μg/m3) and $PM_1/PM_{2.5}$ ratio of the four seasons.

**Season**	**Spring**			**Summer**			**Fall**		
	PM1	$PM_{2.5}$	PM1/PM2.5	PM_1	$PM_{2.5}$	PM1/PM2.5	PM_1	$PM_{2.5}$	PM1/PM2.5
Summer	17.18**	2.56	0.083**						
Fall	9.68*	3.41	0.071**	26.85**	0.45	0.013			
Winter	34.66**	7.82**	0.11**	51.84**	8.76**	0.024**	24.98**	7.91**	0.036**

Note：*p < 0.05; **p < 0.01
